# Using Human iPSC-Derived Neurons to Model TAU Aggregation

**DOI:** 10.1371/journal.pone.0146127

**Published:** 2015-12-31

**Authors:** An Verheyen, Annick Diels, Joyce Dijkmans, Tutu Oyelami, Giulia Meneghello, Liesbeth Mertens, Sofie Versweyveld, Marianne Borgers, Arjan Buist, Pieter Peeters, Miroslav Cik

**Affiliations:** Janssen Research & Development, a division of Janssen Pharmaceutica N.V, Beerse, Belgium; CSIC/Universidad Autonoma Madrid, SPAIN

## Abstract

Alzheimer’s disease and frontotemporal dementia are amongst the most common forms of dementia characterized by the formation and deposition of abnormal TAU in the brain. In order to develop a translational human TAU aggregation model suitable for screening, we transduced TAU harboring the pro-aggregating P301L mutation into control hiPSC-derived neural progenitor cells followed by differentiation into cortical neurons. TAU aggregation and phosphorylation was quantified using AlphaLISA technology. Although no spontaneous aggregation was observed upon expressing TAU-P301L in neurons, seeding with preformed aggregates consisting of the TAU-microtubule binding repeat domain triggered robust TAU aggregation and hyperphosphorylation already after 2 weeks, without affecting general cell health. To validate our model, activity of two autophagy inducers was tested. Both rapamycin and trehalose significantly reduced TAU aggregation levels suggesting that iPSC-derived neurons allow for the generation of a biologically relevant human Tauopathy model, highly suitable to screen for compounds that modulate TAU aggregation.

## Introduction

Several sporadic and familial neurodegenerative disorders are characterized by the formation and deposition of abnormal filamentous proteins in the brain. In Tauopathies like Alzheimer’s disease (AD) and frontotemporal dementia (FTD), the microtubule binding protein TAU is hyperphosphorylated and misfolded in the neurons, leading to neuronal death and cognitive decline (reviewed in [1]). The physiological role of TAU, primarily found in axons, is the polymerization and stabilization of the microtubules and the regulation of axonal transport [2, 3]. In the adult human brain, 6 TAU isoforms are expressed by alternative mRNA splicing of exons 2, 3 and 10 of the *MAPT* gene. At embryonic stages and during development, only the shortest 0N3R isoform is expressed. In contrast, all 6 isoforms are expressed in the adult brain with lower phosphorylation levels than in the fetal brain [4, 5]. However, under pathological conditions like AD and FTD, hyperphosphorylated and aggregated forms of TAU are accumulated in the neurons [6–9], which ultimately leads to neurodegeneration. Several point mutations in exon 10 and mutations affecting exon 10 splicing have been associated with an increased risk for FTD [4, 10, 11].

Various cellular TAU seeding models have been developed to screen for compounds that reduce TAU aggregation [12–14]. Nevertheless, it remains challenging to develop new compounds into effective medicines, partly due to the lack of translational human neuronal models. Recently, a human iPSC-derived 3D model for AD was developed showing TAU aggregation after extended culturing periods [15], making this model unsuitable to screen for compounds that eliminate TAU aggregates. Here we describe a novel human iPSC-derived 2D TAU aggregation model suitable for screening.

## Materials and Methods

### Cell culture, transduction and treatments

iPSC0028 (Sigma) were cultured feeder free (Matrigel) in MW6 plates with mTeSR1 medium and passaged with EDTA. IPSC were differentiated in house or at Axol Biosciences using dual a SMAD inhibition protocol [16].

Neural progenitor cells in MW6 plates were transduced with AAV6-syn1-TAU-P301L or AAV6-syn1-TAU-WT (Dr. Sebastian Kügler, Dept. of Neurology, University Medicine Goettingen, Germany) at MOI 100 with final plating of the cells 24 hours later in PLO/laminin coated MW96 plates.

Rapamycin was dissolved in DMSO while trehalose (both Sigma) was dissolved in culture medium. Compounds were added to the cell culture medium 3 hours before K18 (6,25 nM).

### Preparation of human brain extracts

Human brain tissue was obtained from the Newcastle Brain Tissue Resource at Newcastle University which is a Human Tissue Authority licensed Research Tissue Bank following ethical approval by the National Research Ethics Service. All donations were obtained with fully informed consent following an NRES approved protocol.

Brain samples were cut into in small pieces in frozen state. A 10-fold volume of ice-cold homogenization buffer (10 mM Tris, 150 mM NaCl, pH 7,4, filter: 0,22 μm + EDTA-free protease inhibitors) was added to the frozen brain samples (10% w/v). After homogenization (Teflon potter, B.Braun 30 cc), samples were ultra-centrifuged (27000 x g/19500 RPM—10' - 4°C; Beckman 60 Ti rotor- pre cooled). The supernatant was frozen at -80°C.

### Preparation of K18

Monomeric TAU K18-P301L (40 μM, N and C-terminal myc-tagged) was mixed with 40 μM of heparin, 2mM DTT and 100 mM sodium acetate buffer (pH of 7.0) and incubated at 37°C for 48–72 hours. Afterwards, the mix was centrifuged (100.000g, 1 hour at 4°C), the supernatant discarded and the pellet resuspended in the same final volume of sodium acetate. K18 was freshly sonicated before use (60 cycles of 2 second pulses).

### Quantitative RTPCR

Cells were lysed with RLT buffer + 1% β-mercaptoethanol. RNA was extracted using the RNeasy mini kit (Qiagen) followed by cDNA preparation using SuperScript® III (Life Technologies). The following Taqman assays to detect total *TAU*, 3R and 4R *TAU* isoforms and PGK1 housekeeping gene were purchased (Life technologies): Hs00902194_m1, Hs00902192_m1, Hs00902312_m1 *and* Hs99999906_m1

### SDS PAGE, Native PAGE, Sarkosyl extraction and Western Blot

To detect 3R and 4R TAU isoforms, cells were lysed in RIPA supplemented with protease and phosphatase inhibitors (HALT®; Invitrogen). Proteins were loaded on 4–12% Novex Bis-Tris gels for SDS-PAGE.

To detect TAU aggregates, Blue Native PAGE [17] was performed after lysis of cells in PBS + 0.05% Triton X-100. Protein samples were mixed with Native Page sample buffer and loaded onto a Native Page gel (3–12%) under non-reducing conditions.

For Sarkosyl extraction, cells (+/- 300 000 cells) and brain extracts were lysed and diluted in buffer containing 10 mM Tris, 800 mM NaCl, 1 mM EGTA and 10% sucrose (final concentrations) with protease and phosphatase inhibitors (pH 7.4). Sarkosyl (1% final concentration) was added before ultracentrifugation at 180.000 x g (Beckman TLA-100 rotor) for 1 hour at RT. This centrifugation step was repeated after washing of the insoluble pellet with buffer H (with Sarkosyl1%). Finally, the insoluble pellet was resuspended in TBS/Tween diluted sample buffer.

All gels were blotted on PVDF using the I-blot system. After blocking, HT7 (Thermo Fisher), AT8 (Innogenetics) or 4R TAU primary antibodies (Millipore) were incubated overnight at 4°C. Detection was done with HRP-labeled secondary antibodies (GE Healthcare) via West Dura® or Femto® enhanced chemiluminiscence (Thermo Scientific) kits. Blots were stripped and reprobed with β-actin (Sigma) as loading control.

### Immunostaining

Cells were fixed for 15’ with 4%PFA/4%sucrose in PBS, washed and permeabilized with Triton-X100 (0.25%) in TBS. For TAU aggregates, 1%Triton-X100 was added to the fixative to remove monomeric proteins. After 30’ blocking, cells were incubated overnight at 4°C with following primary antibodies: mouse anti-β3 tubulin, mouse anti-PAX6 (both Covance), chicken anti-MAP2 (Aves), rabbit anti-Tbr1, rat anti-Ctip2, mouse anti-Nestin (all Abcam), rabbit anti-vGlut2, rabbit anti-vGAT (both Synaptic Systems), mouse anti-OCT4, mouse anti-HuC/D (both Invitrogen), mouse anti-Nanog, mouse anti-RD4 (both Millipore), mouse anti-AT8 (Innogenetics) or AT8 conjugated with Alexa 568. The next day, cells were washed and incubated for 1 hour at RT with Alexa secondary antibodies (Life Technologies). DAPI was used to stain the nuclei. Images were taken with OPERA or CV7000 high content readers or the Leica fluorescence microscope.

### Electrophysiology

IPSC-derived neurons were co-cultured for 5 weeks with human primary astrocytes (ScienCell) on coverslips. The perfused extracellular solution (125 mM NaCl, 25 mM NaHCO_3_, 1.25 mM NaH_2_PO_4_, 3 mM KCl, 2 mM CaCl_2_, 1 mM MgCl_2_, 25 mM glucose and 3 mM pyruvic acid. pH adjusted to 7.2–7.4 with NaOH) was maintained at 35°C (95%O_2_, 5%CO_2)_. Intracellular solution was 135 mM potassium gluconate, 7 mM NaCl, 10 mM HEPES, 2 mM Na_2_ATP, 0.3 mM Na_2_GTP and 2 mM MgCl_2_ (pH 7.2–7.4). For Current clamp, cells were injected with current for a holding of -65mV. 20pA steps were applied to evoke spiking with an interval of (-40 to 100pA). For voltage clamp, cells were held at -65mV, currents were injected to increase the voltage steps by 20mV (Range of -80 to +30mV). Spontaneous EPSC were recorded at -65mV with 50μm Picrotoxin (PTX). Traces were acquired with Patchmaster® and analyzed with Fitmaster®

### AlphaLISA

Cells in 96w plate were lysed in 40μ/well RIPA buffer with protease-and phosphatase inhibitors (Roche). After 20–30 minutes of gentle shaking at RT, 5μl sample was mixed with 20 μl biotinylated and acceptor bead-conjugated antibodies in OptiPlate-384 (all Perkin Elmer). After 2 hours of incubation at RT, 25μl of Streptavidin donor beads were added at RT for 30 minutes followed by detection with the Envision plate reader. Raw values were normalized to transduced (no fibril) control samples per plate.

### CellTiter-Glo®

To measure cell health, 5 μl lysate was mixed with 5 μl of CellTiter Glo ® mixture in black low volume plates (Proxiplate™-384 Plus) for 30 minutes followed by luminescence detection using the Envision plate reader.

### Statistics

Data are represented as mean ± SEM unless specified otherwise. Student’s T-TEST was used to compare two groups while 1-way or 2-way ANOVA followed by Tukey’s or Dunnet’s PostHoc tests were used to compare more than 2 groups.

## Results

### Differentiation of hiPSC into cortical neurons and efficient transduction with AAV-TAU-P301L

**Fig 1:**
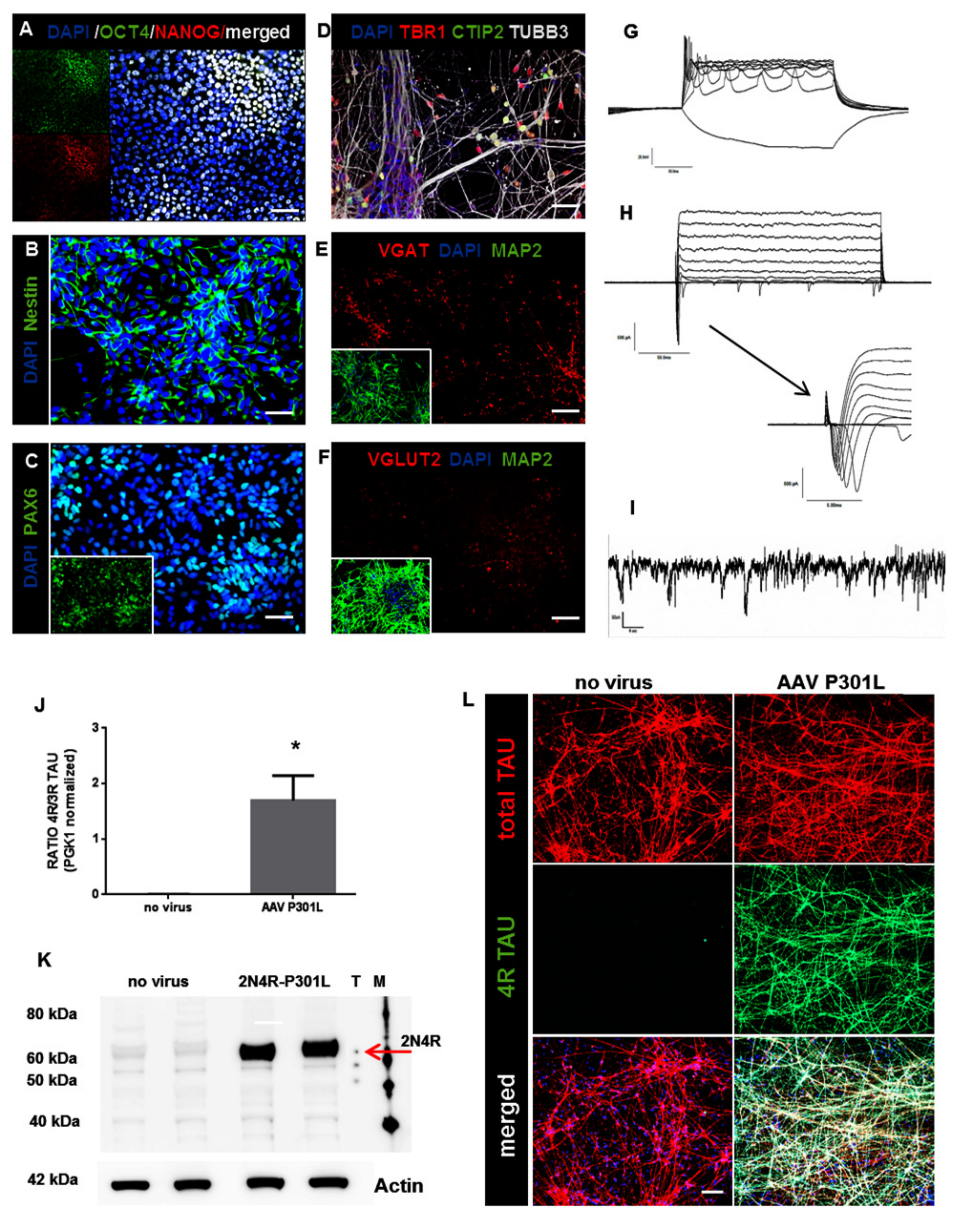
Differentiation of hiPSC into cortical neurons and efficient transduction with AAV-TAU-P301L (A) Immunostaining for OCT4 and NANOG shows that iPSC0028 is pluripotent. Scale bar represents 50μm. (B-C) Immunostaining for Nestin and PAX6 revealing NPC stage at DIV25. Scale bar = 50μm for both. (D-F) Immunostaining on DIV70 visualizes the neuronal marker TUBB3 and cortical markers TBR1 and CTIP2 (D) as well as the dendritic marker MAP2 (E-F) together with either vGLUT2 (E) or vGAT (F). Scale bar = 25μm. (G-I) Representative traces of intrinsic neuronal properties of DIV70 neurons showing evoked responses in current clamp (G) as well as sodium and potassium currents (H) in voltage clamp (n = 13 cells). (I) Example of spontaneous EPSCs recorded at a holding of -65mV in the presence of 50μM PTX in voltage clamp mode. (J) Quantitative RTPCR data showing that transduced neurons express both 3R and 4R *TAU* mRNA, represented by an increased 4R/3R *TAU* ratio compared to non-transduced control cells (P = 0.04; n≥3 from different experiments). Values were normalized to PGK1 before analyses. * P<0,05 (K) Western Blot with a 4R TAU specific antibody depicts 2N4R TAU bands, only in transduced (2N4R-P301L) cells. TAU ladder and marker for band sizes are represented by (T) and (M) respectively. (L) Immunostaining with 4R TAU specific antibody confirms the presence of the 2N4R P301L TAU on the cellular level, only in transduced neurons, while total TAU (red) is present also in control neurons. Scale bar = 25 μm. DAPI stains the nuclei

In this study, we used induced pluripotent stem cells (iPSC) derived from healthy donors. Immunostaining for the pluripotency markers OCT4 and NANOG reveals nuclear expression of both transcription factors (Fig 1A). Further differentiation into cortical neural precursor cells (NPC’s) and immunostaining for PAX6 and Nestin around DIV25 (Fig 1B and 1C) confirms the NPC stage. At this point, NPC’s were either frozen or further differentiated into cortical neurons [16]. Neural identity of the cells around DIV70 is confirmed by immunostaining for the neuronal markers TUBB3 and MAP2 (Fig 1D–1F). Furthermore, TBR1 and CTIP2 staining reveals a cortical identity of the neurons while vGAT and vGLUT2 suggest the presence of both GABAergic and glutamatergic subtypes (Fig 1E and 1F). Western Blot with specific antibodies against 3R and 4R TAU shows that around DIV90 only the embryonic 0N3R TAU isoform is present (S1A Fig). To assess the functionality of the neurons, NPC’s were co-cultured with human astrocytes. Using current clamp, single or multiple action potentials were evoked in 85% of the neurons (n = 13) and with voltage clamp, around 85% of patched neurons had measurable sodium at -20mV (-1.283 ± 0.075nA) and all cells showed potassium currents at 40mV (1.361 ± 0.062nA). Moreover, spontaneous excitatory activity was observed at -65mV confirming functionality and network activity of the neurons [16] (Fig 1G–1I).

To develop a translational TAU aggregation model, we used Adeno-associated virus (AAV) technology to transduce the longest human TAU isoform (2N4R) with P301L mutation into our NPC’s. Four weeks after further differentiation, the presence of the transgene was assessed. Both 3R and 4R *TAU* mRNA is expressed in transduced neurons while only 3R *TAU* mRNA is present in control neurons, leading to an increased 4R/3R ratio in transduced cells (Fig 1J) without significantly changing total *TAU* mRNA and protein levels (S1B and S1C Fig). This might be due to down regulation of endogenous 3R Tau when exogenous 4R TAU is expressed, resembling what has been described during brain development and maturation of fetal and iPSC-derived neurons [5, 18]. Presence of the transgene protein is confirmed using Western Blot (Fig 1K) and immunostaining with a 4R TAU specific antibody (Fig 1L).

### Evaluation of AlphaLISA technology to detect TAU aggregation and phosphorylation in human brain samples

To detect TAU aggregation and phosphorylation, often time consuming and labor intensive ELISA and Western Blot techniques are used after extractions [12, 19] making them less suitable for high throughput screening. Therefore, we evaluated AlphaLISA technology to measure TAU phosphorylation and aggregation as well as total TAU levels in human brain samples. Initial optimizations (not shown) were performed on HEK293 cells and primary rodent neurons overexpressing human TAU-P301L [20].

To allow the detection of aggregates, the monoclonal JRF/hTAU/10 antibody (further referred to as hTAU10) was conjugated to both acceptor beads and biotin. Since monomeric TAU has only one hTAU10 epitope, at least a dimer or more is needed for both acceptor bead-conjugated and biotinylated antibodies to bind and yield a signal upon excitation, independent of the phosphorylation status of the aggregates [20]. The same approach was followed using the phospho-TAU antibody AT8 (pSer202/Thr205) [21]. In this assay only phosphorylated TAU aggregates is detected. Finally, overall TAU phosphorylation at the AT8-epitope can be assessed using biotinylated AT8 in combination with acceptor-bead conjugated hTAU10 (AT8/hTAU10) and total TAU levels can be assessed when a biotinylated HT7 antibody is combined with acceptor-bead conjugated hTAU10 (HT7/hTAU10) as different epitopes are recognized by these 2 total TAU antibodies.

**Fig 2:**
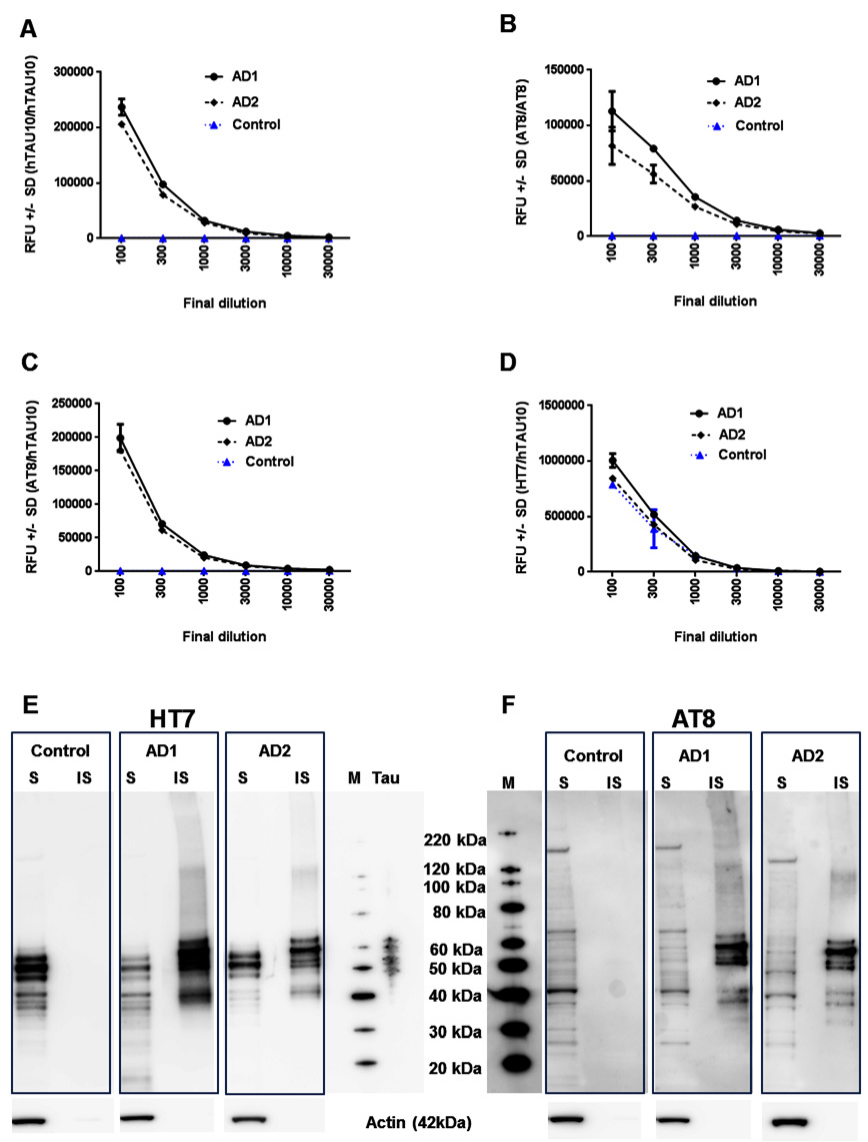
AlphaLISA optimizations on human brain extracts for total TAU, TAU aggregation and phosphorylation. **(A, B)** AlphaLISA on 2 different AD brain extracts show high hTAU10 (A) and AT8 (B) TAU aggregation signals compared to control brain samples. **(C, D)** AT8/hTAU10 (C) AlphaLISA on these AD brain extracts reveals high levels of phosphorylated TAU compared to control brain samples while both AD and control brain extracts display high HT7/hTAU10 (D) levels. Decreasing signals with increasing dilutions suggest no hooking of the samples. Representative curve of 1 experiment with 2 technical replicates is shown as RFU (relative fluorescence units) ± SD. **(E, F)** Western Blot on soluble (S) and insoluble (IS) fractions of control and AD brain extracts after Sarkosyl extraction shows HT7-positive (E) and AT8-positive (F) bands only in the Sarkosyl insoluble pellets of both AD patients, confirming the presence of TAU aggregates. M represents Magic Marker (band sizes) and T represents TAU ladder with all 6 TAU isoforms. All experiments have been confirmed at least twice.

Human brain extracts from 2 AD patients and 1 healthy control (Newcastle Brain Tissue Resource, Newcastle University; S1 Table) were obtained for extended validation of these TAU-quantification assays in a human setting. Brain extracts from both AD donors show high hTAU10 and AT8 TAU-aggregation signals (Fig 2A and 2B) as well as high TAU phosphorylation levels (Fig 2C) in comparison to control brain extract, while total TAU is expressed to a similar level in all samples tested (Fig 2D). Additionally, after Sarkosyl fractionation of both control and AD brain extracts, only the Sarkosyl insoluble fractions of the two AD brains reveal HT7-positive and AT8-positive bands (Fig 2E and 2F), confirming our AlphaLISA results.

### K18 seeding induces TAU aggregation in human TAU-P301L neurons

Due to the lack of spontaneous aggregation 4 weeks after final plating, we seeded our transduced human neurons with K18 (P301L), which has been shown to facilitate TAU aggregation in cellular and primary neuron model systems [14, 19].

**Fig 3:**
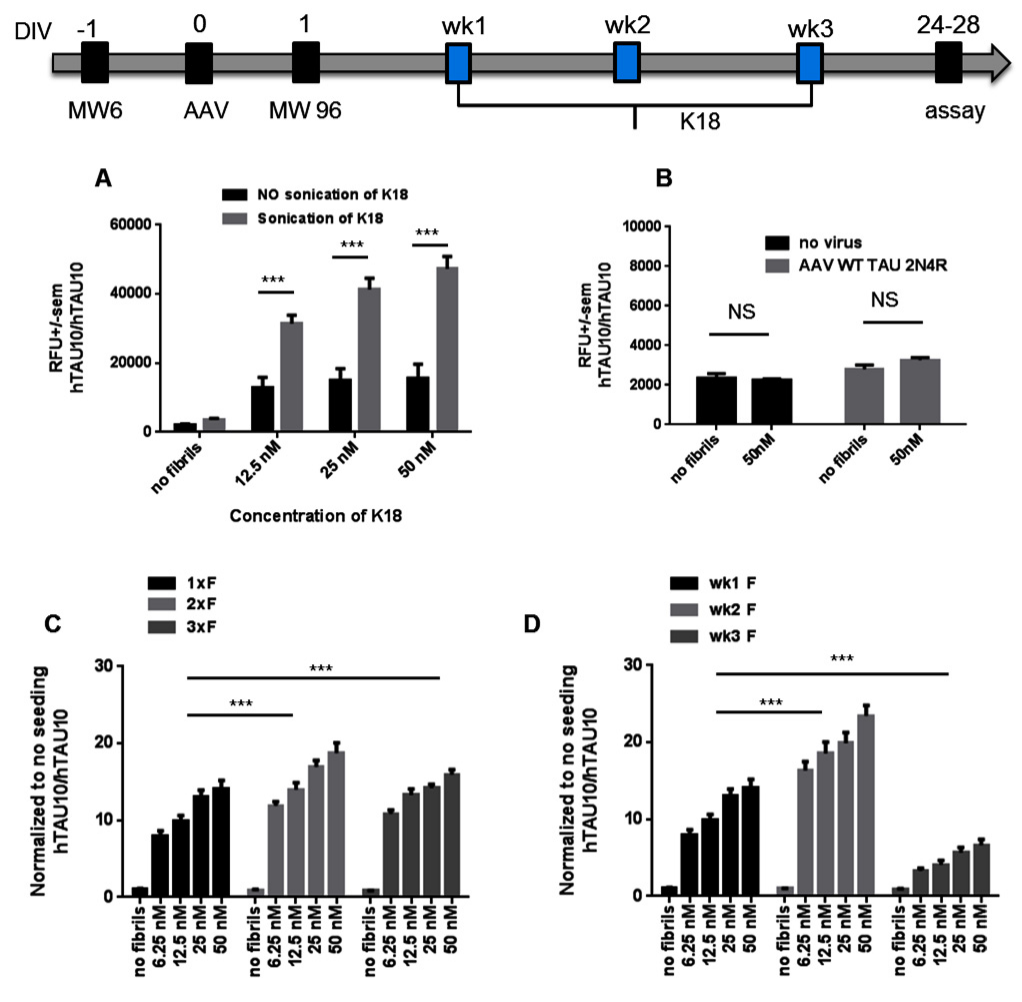
Optimization of dynamic range of hTAU10 aggregation AlphaLISA. **(A)** K18 sonication significantly improves seeding potency (P<0.001, n≥3 independent experiments). Seeds were added at week 1. In all further experiments, sonicated K18 is used. **(B)** K18 seeding does not induce aggregation in control (no virus) and WT virus (AAV WT TAU 2N4R) transduced neurons (P = NS; n≥3 independent experiments). **(C)** Weekly repeated K18 seeding of K18 significantly increases the dynamic range (P<0.001 for both 1xF *versus* 2xF (wk 1+2) and 1xF *versus* 3xF (wk1+wk2+wk3); n≥3 independent experiments). **(D)** Finally, also the timing of seeding has an effect on the aggregation potency. Addition of K18 at week 2 (wk2) significantly increases TAU aggregation compared to week 1 (P<0.001, n≥3 independent experiments) while addition of fibrils at week 3 shows significantly less aggregation (P<0.001; n≥3 independent experiments) probably due to the shorter (1 week) K18 incubation period before AlphaLISA. ***P<0,001; 2-way-ANOVA with Dunnett’s post hoc.

**Fig 4:**
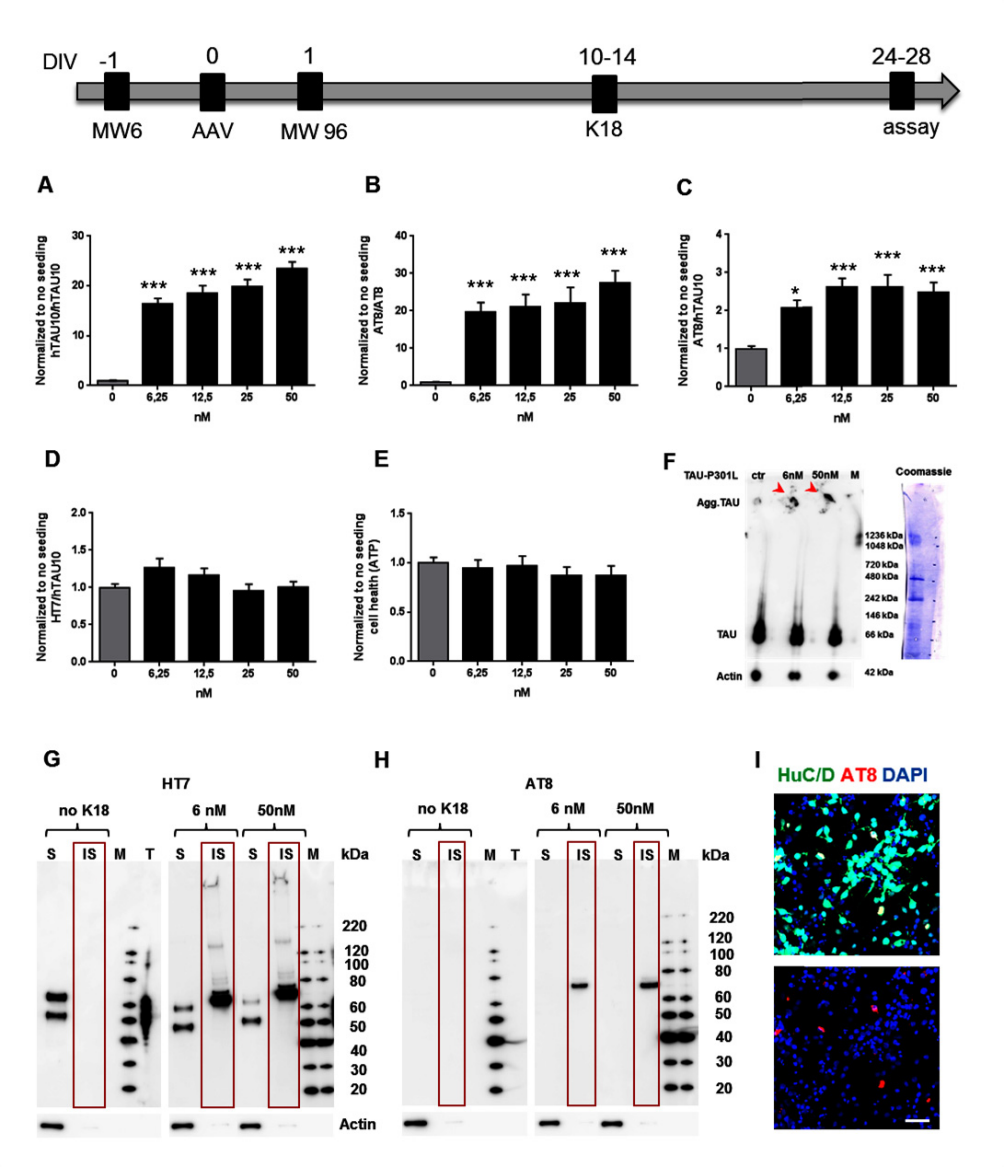
K18 seeding induces TAU aggregation and hyperphosphorylation in human TAU-P301L neurons. Optimal timelines are shown for AAV transduction, 96w final plating, K18 seeding and final assay. **(A, B)** AlphaLISA data show that K18 seeding induces an increase in both hTAU10 (P<0,001; A) and AT8 (P<0,001; B) TAU aggregation assays. **(C, D)** AlphaLISA results demonstrate around 2-fold increase in TAU phosphorylation (AT8/hTAU10; P<0,001; C) while total TAU levels remain unchanged (HT7/hTAU10; P = NS; D). **(E)** CellTiter-Glo® results showing that general cell health is unaffected after K18 addition (P = NS). For all assays in **(A-E)**: *** P<0,001; 1-way ANOVA with Tukey’s post hoc; n≥3 independent experiments. **(F)** Representative blot of Native PAGE followed by Western blot showing two monomeric HT7-positive TAU bands (around 66kDa) in all conditions. Notably, non-migrated HT7-positive TAU proteins (>1236kDa) in K18-seeded samples suggest the presence of TAU aggregates. **(G, H)** Representative Western blots after Sarkosyl extraction showing soluble (S) and insoluble (IS) fractions after blotting with antibodies against total TAU (HT7; G) and hyperphosphorylated TAU (AT8; H). Aggregates are only present in the insoluble pellet after addition of 6nM or 50nM of K18 fibrils. Note the presence of monomeric 3R and 4R TAU protein in the soluble fraction. **(I)** Immunostaining for AT8 and neuronal HuC/D after 1%Triton/PFA fixation, to remove monomeric TAU, reveals AT8-positive neurons after K18 seeding. Scale bar = 25μm.

In a first set of experiments we focused on the optimization of our culture and K18 seeding conditions in combination with our hTAU10 aggregated TAU AlphaLISA to identify a suitable dynamic range for screening purposes. More specifically, different concentrations of K18 fibrils were added to the culture medium between 1 and 3 weeks after final plating and AlphaLISA was performed at DIV 28. Our results demonstrate that K18 seeding 1 week after plating induces an increase in hTAU10/hTAU10 signal, which is significantly higher when K18 is freshly sonicated (Fig 3A). Note that K18 fibrils do not induce aggregation in control neurons or NPCs transduced with wild type human TAU (Fig 3B). Also weekly re-seeding (Fig 3C) or adding K18 one week later (Fig 3D) significantly improved the dynamic range. From these results we concluded that the most robust and reproducible readout was achieved when sonicated seeds were added 2 weeks after final plating with AlphaLISA performed 2 weeks later, resulting in reproducible TAU aggregation levels within 4 weeks (Fig 4A and S2 Fig). Under these conditions, there is a similar increase in AT8 positive aggregates (Fig 4B and S2 Fig). Furthermore, we observe a 2-fold increase in general TAU phosphorylation compared to no seeding (Fig 4C and S2 Fig) while total TAU levels (Fig 4D and S2 Fig) and total ATP levels reflecting general cell health (Fig 4E) remain unchanged.

To confirm the presence of TAU aggregates, we performed native PAGE [17] followed by Western blot. Our results reveal two HT7-positive TAU bands around the size of monomeric TAU (arrow in Fig 4F), in all conditions, likely corresponding to 3R and 4R TAU. Furthermore, larger sized (>1200kD) TAU aggregates visible at the top of the gel (arrowheads) are detected in K18-seeded samples only, suggesting the presence of TAU aggregates (Fig 4F). Also Western Blot on soluble and insoluble fractions after Sarkosyl extraction shows HT7-positive and AT8-positive bands only in the Sarkosyl insoluble pellet of K18 seeded samples, reinforcing the presence of aggregates (Fig 4G and 4H). Finally, TAU aggregates are visualized at the cellular level by AT8 staining in combination with the neuronal marker HuC/D, only in K18-treated neurons (Fig 4I; non-treated cells not shown), after 1% Triton/PFA fixation to remove soluble TAU.

### Autophagy inducers reduce TAU phosphorylation and aggregation in TAU-P301L neurons

**Fig 5:**
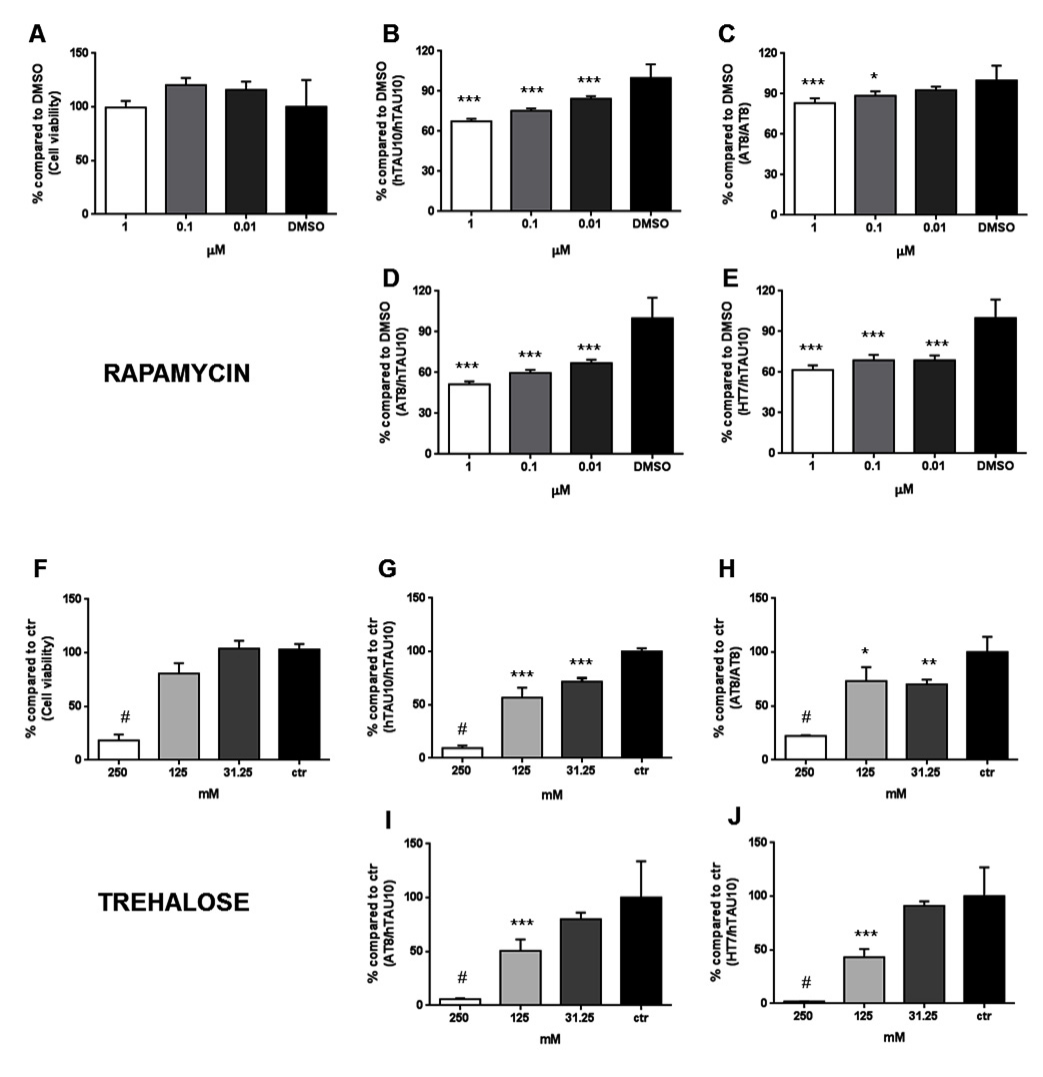
Model validation: autophagy inducers reduce TAU hyperphosphorylation and aggregation in TAU-P301L neurons. **(A)** CellTiter-Glo® data showing that rapamycin is not toxic (P = NS). **(B, C)** Rapamycin dose-dependently reduces hTAU10 (P<0,001; B) and AT8 aggregated TAU measured with AlphaLISA (P = 0,025 at 10 nM and P<0.001 at 1 μM; C) and compared to DMSO. **(D, E)** Also general TAU phosphorylation is reduced (AT8/hTAU10; P<0,001; D) to a similar extent as the reduction in total TAU (HT7/hTAU10; P<0,001; E). **(F)** CellTiter-Glo® results show that trehalose is highly toxic at 250 mM (P<0,001). **(G, H)** AlphaLISA results reveal that trehalose significantly reduces hTAU10 (P<0,001) and AT8 TAU aggregation levels *versus* control (P = 0,006 at 31,5 mM and P = 0,014 at 125 mM). **(I, J)** Only at 125 mM of trehalose, both phosphorylated TAU (P<0,001, I) and total TAU levels (P<0,001, J) are decreased. ***P<0,001; **P<0,01; *P<0,05; ^#^P<0,001 due to toxicity; 1-way ANOVA with Dunnett’s post hoc; n≥3 independent experiments

To validate our assay for screening purposes, we selected two autophagy-inducing compounds rapamycin and trehalose described to reduce TAU aggregation and phosphorylation *in vitro* and *in vivo* [22–25] and tested different concentrations in the human iPSC-derived neuronal TAU aggregation assay. Rapamycin at the concentrations tested appears to be not toxic for the cells (Fig 5A) and induces a concentration-dependent reduction in TAU aggregation (Fig 5B and 5C). At 1 μM we observe an approximately 32% decrease in hTAU10/hTAU10 (Fig 5B) signals while phosphorylated aggregates (AT8/AT8) are reduced by 16% (Fig 5C). Furthermore, phosphorylation at the AT8 epitope is inhibited by almost 50% (Fig 5D). Notably, total TAU is also reduced (Fig 5E). Trehalose shows significant toxicity at 250 mM and a trend at 125 mM (P = NS) (Fig 5F). The lowest tested concentration significantly reduces both hTAU10 and AT8 TAU aggregation Fig 5G and 5H). Only at 125 mM of trehalose, a reduction of TAU phosphorylation and total TAU (Fig 5I and 5J) levels are observed.

## Discussion

In this study, we describe a novel and biologically relevant human neuronal TAU aggregation model by introducing mutant TAU-P301L into healthy iPSC-derived NPC’s with further differentiation into cortical neurons [16]. During initial characterization of our control iPSC-derived cortical neurons, we failed to detect 4R TAU, suggestion that our neurons display an immature phenotype when taking the juvenile 3R TAU-expression as the marker, even after more than 90 days *in vitro*. These results are in line with recent publications showing expression of mature TAU isoforms in iPSC-derived cortical neurons only after extended culturing periods [26, 27]. Therefore, the lack of exon 10 in young cortical neurons might limit the detection of a TAU aggregation phenotype in iPSC-derived neurons from patients with pro-aggregating point mutations in this exon [4, 10, 11]. Although, it has been shown that iPSC-derived neurons from patients with TAU-P301L mutation do show mitochondrial deficits and changed excitability after extended culturing periods [27].

Expression of longest TAU isoform carrying the pro-aggregating P301L mutation in our hiPSC-derived neurons failed to induce spontaneous TAU aggregation confirming previously published iPSC-derived Tauopathy models [27–29]. Therefore TAU aggregation was triggered using preformed aggregates consisting of the TAU-microtubule binding repeat (K18), which has been proven to facilitate TAU aggregation in several *in vitro* and *in vivo* TAU seeding models [19, 30, 31]. Also in our model, a fast and robust TAU aggregation and hyperphosphorylation phenotype was detected, using AlphaLISA technology, the no-wash ELISA alternative that has been widely used in screening campaigns [32, 33]. Remarkably, sonication of K18 significantly increased the dynamic range of the assay suggesting that smaller aggregates have a higher seeding potency.

Activation of autophagy has been shown to protect neurons by the degradation of misfolded proteins (reviewed in [34]). In our hiPSC-derived neurons, both trehalose and rapamycin reduced TAU aggregation and phosphorylation levels as well as total TAU, confirming published data on different cell lines and TAU-P301S mice [22–25]. These results confirm the potency of autophagy inducers to clear TAU aggregates, also in human neurons.

Currently, iPSC-derived neurons might be less suitable for primary screening purposes due to high costs and relatively long cultivation periods. On the other hand, our model could serve as a more relevant biological tool to confirm hits coming from primary TAU aggregation inhibition screens, in which cell lines are used. Furthermore, our model also allows to identify new targets and mechanisms linked with TAU aggregation, potentially leading to new drugs to treat AD and FTD.

## Supporting Information

S1 Fig Control iPSC-derived neurons only express 0N3R TAU and do not increase total TAU levels after transduction with AAV-TAU-P301L. **(A)** SDS-PAGE and Western Blot for 3R and 4R TAU on DIV 90 neurons derived from iPSC0028 reveals that only the 0N3R TAU isoform is detected, while 4R TAU is absent. 0N3R and 0N4R recombinant TAU proteins were added as positive controls, as well as a TAU ladder (rPeptide). A downward shift is seen after dephosphorylation (+λ, in duplo) of the 2 samples compared to non-treated samples (-λ) (RD3 = 3R and RD4 = 4R TAU antibody). **(B)** Quantitative RTPCR on control and AAV TAU (P301L) transduced cells shows no difference in total *TAU* mRNA levels, four weeks after transduction (P = NS, n = 3, T-TEST, normalized to PGK1). **(C)** HT7/hTAU10 AlphaLISA results showing no difference in total TAU levels 4 weeks after transduction (P = NS, n = 3, T-TEST, normalized to no virus)
(EPS)

S2 Fig AlphaLISA on iPSC-derived neurons for total, aggregated and hyperphosphorylated TAU. In complement with Figs 3 and 4 of the main paper, K18-treated samples were diluted (1/3 dilutions) and tested in our 4 AlphaLISA assays. Note that ‘pure sample’ is depicted as final 1/10 dilution (5μl volume of pure sample + beads mixture volume of 45 μl). Both aggregating TAU assays **(A and B)** as well as phosphorylated **(C)** and total TAU **(D)** assays show high signals that decrease with increased dilutions, suggesting that we are measuring in a linear range without hooking of the sample. RFU (relative fluorescence units) ± SD. Graph of 1 representative experiment with 2 technical replicates is shown. Results have been confirmed at least twice.
(EPS)

S1 Table Information on AD brain and control brain donors. Donor information of AD and control brain extracts that have been used to validate our AlphaLISA assays. This table is related with Fig 2 of the main figures.
(PDF)
