# Supplementary material for: Using Human iPSC-Derived Neurons to Model TAU Aggregation
Source: PLoS One. 2015 Dec 31;10(12):e0146127. doi: 10.1371/journal.pone.0146127 (PMC4697850; doi:10.1371/journal.pone.0146127)
Supplement: S1 Table — Donor information of AD and control brain extracts that have been used to validate our AlphaLISA assays. This table is related with Fig 2 of the main figures. (PDF) [file pone.0146127.s003.pdf]

Sample	ID	gender	Age	Post- mortem (h)	BRAAK	Tissue
**AD1**	74 92	female	67	47	6	Parietal temporal
**AD2**	924	female	81	56	6	Parietal temporal
**Control**	958	female	48	48	0	Parietal temporal
